# Robustness in Older Adults: A Concept Analysis Using Rodgers' Evolutionary Approach

**DOI:** 10.1111/nhs.70349

**Published:** 2026-05-04

**Authors:** Yoosun Yang, Jun‐Ah Song

**Affiliations:** ^1^ College of Nursing Korea University Seoul Republic of Korea; ^2^ Institute on Aging Korea University Seoul Republic of Korea

**Keywords:** aging, concept analysis, gerontology, older people, robustness

## Abstract

As the number of healthy older adults increases, robustness has emerged as an important concept to describe individual differences in adaptability and health outcomes. However, the concept remains inconsistently defined across healthcare disciplines, necessitating conceptual clarification. This study aims to conduct an evolutionary concept analysis of robustness in older adults. An evolutionary concept analysis method was conducted. Four healthcare databases (PubMed, CINAHL Complete, EMBASE, Scopus) and Google Scholar were systematically searched without publication year restrictions, excluding gray literature. Data were extracted and analyzed to identify attributes, antecedents, consequences, and related terms. Analysis of 31 studies revealed three attributes: multidimensional vitality, resilient stability, and functional autonomy. Antecedents include socioeconomic resources, beneficial lifestyle behaviors, and supportive social networks, while consequences encompass enhanced quality of life, reduced healthcare needs, and decreased mortality. Comparison with related terms confirmed robustness as distinct from successful aging, non‐frailty, intrinsic capacity, and resilience. This study established conceptual boundaries for robustness, enabling healthcare professionals to move beyond age‐based approaches toward individualized assessment and care strategies, providing a theoretical framework for developing measurement tools and implementing personalized interventions.

## Key Points

- Robustness in older adults is characterized by three distinct attributes: multidimensional vitality (physical, emotional, cognitive, and social vigor), resilient stability (adaptive capacity to maintain health despite challenges), and functional autonomy (independence in daily activities and decision‐making).
- Unlike related concepts such as successful aging, non‐frailty, intrinsic capacity, and resilience, robustness in older adults emphasizes dynamic adaptation and recovery processes reflecting a contemporary view of aging as continuous growth and adaptation.
- Healthcare professionals should shift from age‐based approaches to individualized assessment and interventions based on robustness levels, requiring the development of comprehensive assessment tools that integrate all three robustness attributes for evidence‐based geriatric care.

## 1 Introduction

According to the World Population Prospects 2024 data, the population aged 80 and over is expected to exceed the infant population under 1 year of age by the mid‐2030s, and by the late 2070s, the population aged 65 and over is projected to reach 2.2 billion, surpassing the population under 18 years of age (United Nations 2024). Alongside this demographic shift, the number of older adults aging healthily is also increasing. Thanks to advances in medical technology (Soffer et al. 2024) and the widespread adoption of preventive healthcare (Wang and Wang 2021), 81% of older adults worldwide maintain high functional capacity, and 85% possess adequate intrinsic capacity (Rudnicka et al. 2020).

As the number of healthy older adults increases, researchers have begun to focus on individual differences in health outcomes and adaptability within this population (Laird et al. 2018; Musich et al. 2022). In particular, growing attention is being paid to the remarkable resilience and adaptive capacity displayed by some older individuals despite being of the same chronological age (Gijzel et al. 2019; Górska et al. 2021). In this context, the term “robustness” is frequently used to describe these differences (Costa‐de Lima et al. 2015; Maia et al. 2020).

Several studies have examined robust older adult populations, with findings demonstrating that these individuals exhibit significantly lower rates of multimorbidity and disability (Chang et al. 2019; Mehrabi and Béland 2021; Stolz et al. 2024). Additionally, robust older adults show marked advantages when undergoing aggressive medical interventions, including chemotherapy and surgical procedures (Rothrock et al. 2019; Xu et al. 2021). These findings suggest that robust older adults constitute a clinically distinct population group, which provides evidence for the development of personalized geriatric care and targeted healthcare services by highlighting the need for medical decision‐making that considers robustness levels.

While robustness has emerged as an important concept in older adult population, its definition and application remain inconsistent across studies (Hoeksema et al. 2017; Md Fadzil et al. 2023; Rodin 2017). For instance, when “robust older adults” groups are used as control groups in frailty‐related research, robustness is frequently defined merely as the absence of frailty indicators (Mehrabi and Béland 2021; Stolz et al. 2024). Frailty, a multidimensional condition characterized by functional, social, and cognitive decline (Navarrete‐Villanueva et al. 2021), has been identified as a primary predictor of vulnerability in older adults (Rocha et al. 2025). In contrast, other studies conceptualize robustness as a distinct concept of frailty, emphasizing the unique adaptive capacity and recovery abilities inherent in older adults (Costa‐de Lima et al. 2015; Garfein and Herzog 1995). These divergent approaches underscore the need for conceptual clarification to achieve a consistent understanding and application of the robustness concept. Accordingly, this study aims to analyze the concept of ‘robustness in older adults’ to elucidate its attributes, antecedents, and consequences, thus establishing a clearer conceptual foundation for research and practice with older adults.

## 2 Methods

This study explored the concept of robustness in older adults through concept analysis—a method used to clarify and deepen understanding of concepts by synthesizing existing literature (Tofthagen and Fagerstrøm 2010). As robustness is widely used across multiple disciplines and has evolved conceptually over time, this study employed the evolutionary concept analysis method proposed by Rodgers and Knafl (2000), which is grounded in the view that concepts are dynamic and change over time (Toulmin 1972). Rodgers and Knafl (2000) described the purpose of this method as identifying “a current consensus or ‘state of the art’ regarding the concept” (p. 83) and examining “areas of agreement and disagreement across disciplines, change over time” (p. 95), thus providing a foundation for further development of the concept.

The evolutionary method of concept analysis involves multiple activities often conducted simultaneously rather than as sequential steps, including identifying the concept and its surrogate terms, selecting an appropriate sample for data collection, analyzing the concept's attributes and contextual basis across interdisciplinary and sociocultural dimensions, identifying exemplars, and drawing implications for further concept development (Rodgers and Knafl 2000).

Data collection was conducted across four databases—PubMed, CINAHL Complete, EMBASE, and Scopus—with supplementary searches performed in Google Scholar to identify additional relevant references through ancestry searching and forward citation searching. In accordance with the evolutionary methodology guidelines, an open approach was adopted without publication year restrictions to ensure comprehensive coverage (Rodgers and Knafl 2000). The primary search terms included “robust*,” “old*” or “elder*,” and “agi*,” which were combined using Boolean operators to search for relevant publications (Supporting Table 1). Related terms for robustness in older adults were not predetermined but were identified and analyzed during later stages of the concept analysis process.

The literature selection criteria were as follows: (a) papers published in English, (b) papers with full text available, (c) papers that address and clearly describe the concept of robustness in older adults, and (d) papers that have the potential to explain the attributes, antecedents, consequences, surrogate/related terms, or contextual foundations of the robustness concept in older adults.

**FIGURE 1:**
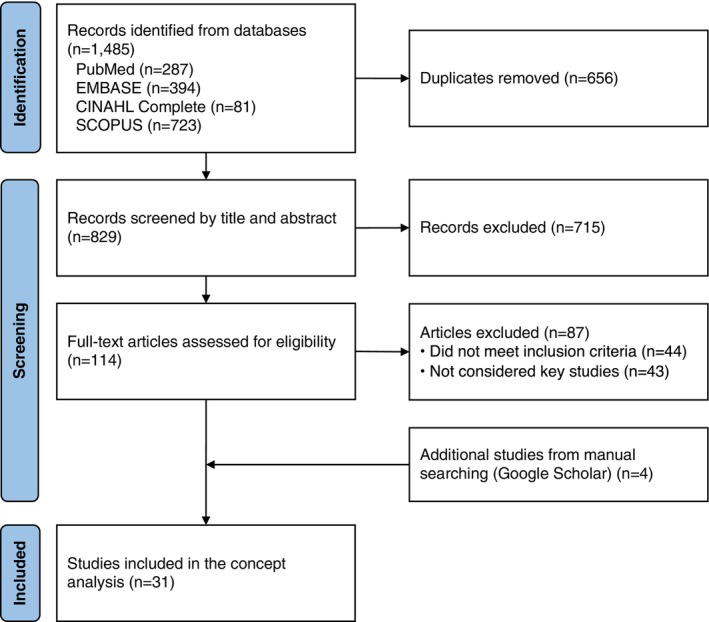
Flow diagram of the literature selection process.

Through a systematic literature selection process, 31 key studies were finally selected from a total of 1485 studies (Figure 1), meeting the evolutionary method's recommendation of a minimum of 30 studies (Rodgers and Knafl 2000).

The selected key studies were comprehensively analyzed using guiding questions according to the evolutionary analysis method (Rodgers and Knafl 2000). The guiding questions included: terms used as substitutes for the concept (surrogate terms), terms that share commonalities with the concept (related terms), relevant preceding events or conditions (antecedents), core characteristics of the concept (attributes), specific examples that illustrate the concept (exemplars), and outcomes resulting from the concept (consequences) (Tofthagen and Fagerstrøm 2010). To enhance reliability, two researchers independently analyzed the literature and reached consensus through regular discussions. Conceptual clarity was considered achieved when consistent patterns in the concept's characteristics appeared repeatedly and the overall relationships among antecedents, attributes, and consequences became evident.

## 3 Results

### 3.1 Context of the Concept

The Oxford English Dictionary defines it as “the condition or quality of being robust (in various senses); sturdiness, hardiness; strength” (Oxford English 2023) and defines robust as “strong and hardy; strongly and solidly built, sturdy; healthy (Oxford English 2025).” Definitions of robustness also have been examined across multiple disciplines, including biology, engineering, management, neuroscience (Table 1). Despite variations in definitions, robustness consistently refers to the ability to maintain stability and functionality despite internal and external disturbances.

**TABLE 1:** Definitions of robustness across various academic disciplines.

Author (year)	Academic field	Definition
Kitano (2004)	Biological	Robustness is a property that allows a system to maintain its functions despite external and internal perturbations.
Baker et al. (2008)	Engineering	Robustness is taken to imply tolerance to damage from extreme loads or accidental loads, although the framework here is applicable to other adverse effects such as sensitivity to human error and deterioration.
Durach et al. (2015)	Management	Robust supply chains, where robustness corresponds primarily with being physically sturdy and being able to retain the same stable situation as before changes occurred.
Ishigami and Klein (2011)	Neuroscience	Robust means that network scores remain significantly different from zero with repeated testing.

However, conceptualizing the concept of robustness to older adults requires a contextually different approach. Definitions of robustness in other disciplines focus on maintaining unchanging stability, whereas aging is a natural process accompanied by biological, psychological, and social changes (Dziechciaż and Filip 2014; Scott 2022). Therefore, defining robustness in older adults merely as “resistance to change” creates a contradiction, as it implies that the aging process itself is a failure or a form of vulnerability. In this context, robustness in older adults is understood differently from other academic fields, with greater emphasis on dynamic adaptation and recovery processes (Choi et al. 2024; Rodin 2017).

Meanwhile, the concept of robustness in older adults has evolved over time. Early gerontology introduced the concept of successful aging as distinct from normal aging (Rowe and Kahn 1987), and during this period, robust aging and successful aging were used interchangeably (Kiyak 2000). A turning point emerged when Fried et al. (2001) developed the concept and measurement tool for frailty. They established robustness as the opposite concept of frailty by classifying older adults without frailty characteristics as robust. This approach was subsequently reflected in the development of various frailty measurement tools (Ensrud et al. 2007; Searle et al. 2008), and robustness and frailty have since often been understood as concepts located at opposite ends of a continuum (Mehrabi and Béland 2021; Mori and Tokuda 2019). However, this opposition should not be understood merely as a static contrast. Fried et al. (2021) proposed in a subsequent study that “robustness, resilience and frailty reflect different points on a continuum of physiological fitness and reserve” (p. 44), emphasizing that the opposition between robustness and frailty should be understood not as a contrast between fixed states but as an opposition within a dynamic process.

As the scope of frailty subsequently expanded beyond physical deficits to include cognitive and social dimensions (Bunt et al. 2017; Kelaiditi et al. 2013), its conceptual “opposite” also had to evolve. Robustness is now understood as a multidimensional concept that encompasses physical, cognitive, and social domains (Hoeksema et al. 2017; Md Fadzil et al. 2023). For example, physical robustness may manifest as preserved gait speed and grip strength (Liu et al. 2019), cognitive robustness as intact working memory and attention (Bu et al. 2021), and social robustness as sustained network size and engagement (Huang et al. 2021).

**FIGURE 2:**
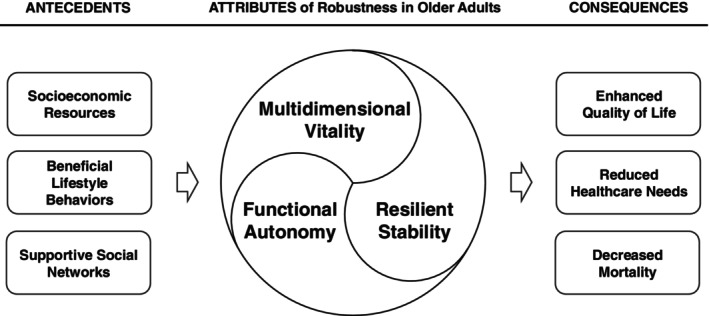
Conceptual model of robustness in older adults.

This study identified the attributes, antecedents, and consequences of robustness in older adults, which are presented as a conceptual model (Figure 2). The specific characteristics of these components are as follows:

### 3.2 Attributes

#### 3.2.1 Multidimensional Vitality

Multidimensional vitality was identified as an attribute in 28 key studies (Table 2). This represents a comprehensive state of vigor encompassing physical, emotional, cognitive, and social aspects. Specifically, the physical dimension included high levels of physical activity such as strength testing, stair climbing, and sensory function (Armi et al. 2008; Chang et al. 2018), while the emotional dimension encompassed low levels of depression and anxiety (Liu et al. 2021; Stolz et al. 2024). The cognitive dimension included the absence of mild cognitive impairment (MCI), high intellectual ability, and good memory (Chen et al. 2022; Kiyak 2000), and the social dimension encompassed active social contact and participation in various social activities (Bakker et al. 2020; Mehrabi and Béland 2021).

**TABLE 2:** Identification of concept analysis through key studies.

Author (year)	Attributes			Antecedents			Consequences		
	Multi‐dimensional vitality	Resilient stability	Functional autonomy	Socioeconomic resources	Beneficial lifestyle pattern	Supportive social networks	Enhanced quality of life	Reduced healthcare needs	Decreased mortality
Kiyak (2000)	✓	✓	✓	✓					
Stolz et al. (2024)	✓	✓	✓	✓					✓
Mehrabi and Béland (2021)	✓	✓	✓	✓		✓			
Liu et al. (2021)	✓	✓				✓	✓		
Armi et al. (2008)	✓	✓	✓						
Xu et al. (2021)	✓	✓	✓						✓
Bakker et al. (2020)	✓	✓	✓		✓				
Chang et al. (2018)	✓							✓	
Mori and Tokuda (2019)	✓	✓			✓		✓		
Chen et al. (2022)	✓	✓	✓				✓	✓	✓
Kim et al. (2019)	✓	✓	✓		✓			✓	✓
Hoeksema et al. (2017)	✓	✓	✓	✓			✓		
Kim et al. (2023)	✓	✓	✓					✓	
Shi et al. (2019)	✓	✓	✓						✓
Md Fadzil et al. (2023)	✓	✓		✓					
Kang et al. (2017)	✓	✓	✓	✓	✓				
Chang et al. (2019)	✓	✓							
Rodin (2017)		✓							
Sanoff and Goldberg (2013)		✓	✓						
Ho and Neo (2021)	✓	✓	✓						
Chang and Wen (2016)	✓	✓		✓	✓	✓	✓		
Brustio et al. (2022)	✓		✓		✓				
Motokawa et al. (2018)	✓	✓	✓		✓				
Maia et al. (2020)	✓	✓	✓	✓	✓	✓			
Noguchi et al. (2022)	✓	✓	✓	✓	✓	✓		✓	
Malek Rivan et al. (2019)	✓		✓	✓	✓	✓			
de Labra et al. (2018)	✓			✓		✓	✓		
Costa‐de Lima et al. (2015)		✓	✓						
Rothrock et al. (2019)	✓	✓							
Choi et al. (2024)	✓	✓	✓		✓		✓		
Garfein and Herzog (1995)	✓		✓	✓		✓			
Total	28	26	22	12	11	8	7	5	5

#### 3.2.2 Resilient Stability

Resilient stability was identified as an attribute in 26 key studies (Table 2). This refers to the ability to maintain stability by adapting and recovering when faced with changes and challenges. Specifically, this encompasses the ability to effectively prevent or delay the onset of diseases, including chronic conditions, throughout the aging process (Choi et al. 2024; Costa‐de Lima et al. 2015; Kim et al. 2023), the capacity to actively engage in and overcome treatment for serious illnesses such as cancer (Rodin 2017; Sanoff and Goldberg 2013; Xu et al. 2021), and the ability to maintain health through reduced polypharmacy and optimized medication management patterns (Choi et al. 2024; Liu et al. 2021).

#### 3.2.3 Functional Autonomy

Functional autonomy was identified as an attribute in 22 key studies (Table 2). This refers to the ability to perform daily activities and make personal decisions either independently or with necessary assistance. Specifically, this includes the ability to independently perform activities of daily living (ADL) and instrumental activities of daily living (IADL) (Brustio et al. 2022; Costa‐de Lima et al. 2015; Malek Rivan et al. 2019), the ability to make active decisions while maintaining control over one's life with minimal care needs and low levels of required assistance (Hoeksema et al. 2017; Kim et al. 2023), and the ability to perform various functional roles including social participation and productive activities (Garfein and Herzog 1995; Kim et al. 2023).

### 3.3 Antecedents

Through concept analysis, three antecedents were identified. First, socioeconomic resources were identified as an antecedent in 12 key studies. Specifically, higher financial stability (Hoeksema et al. 2017; Kang et al. 2017; Kiyak 2000) and educational level (Chang and Wen 2016; Md Fadzil et al. 2023; Noguchi et al. 2022) were observed to be associated with greater robustness in older adults.

Next, beneficial lifestyle behaviors were identified as an antecedent in 11 key studies. Specifically, regular exercise habits (Brustio et al. 2022; Maia et al. 2020; Mori and Tokuda 2019), adequate nutritional status (Malek Rivan et al. 2019; Motokawa et al. 2018), and non‐smoking status (Chang and Wen 2016; Kang et al. 2017) were associated with higher robustness.

Finally, supportive social networks were identified as an antecedent in eight key studies. Specifically, higher levels of social connectivity including family (Chang and Wen 2016; Garfein and Herzog 1995; Liu et al. 2021; Mehrabi and Béland 2021) and more supportive environments (Malek Rivan et al. 2019; Noguchi et al. 2022) were associated with higher robustness.

### 3.4 Consequences

Through concept analysis, three consequences were identified. First, enhanced quality of life was identified as a consequence in 7 key studies. These studies utilized standardized instruments including SR‐12 (Chen et al. 2022), SF‐36 (Liu et al. 2021; Mori and Tokuda 2019), WHOQOL‐BREF (Chang and Wen 2016; de Labra et al. 2018), and EQ‐5D (Choi et al. 2024; Hoeksema et al. 2017), consistently revealing positive associations between robustness and various dimensions of quality of life.

Second, reduced healthcare needs were identified as a consequence in 5 studies. Specifically, robust older adults had lower hospitalization risks (Chang et al. 2018; Chen et al. 2022) and fewer hospitalization cases (Choi et al. 2024). Regarding healthcare service utilization, they incurred the lowest medical costs with the fewest outpatient visits, hospital admissions, and hospital days (Kim et al. 2023). Additionally, robust older adults had a lower likelihood of long‐term care facility admission (Kim et al. 2019).

Last, decreased mortality was identified as a consequence in five studies. According to the research, robust older adults showed lower mortality rates and reduced death risks compared to non‐robust older adults, demonstrating longer overall survival and higher survival rates (Kim et al. 2019; Shi et al. 2019; Stolz et al. 2024; Xu et al. 2021).

### 3.5 Related Terms

This study revealed that “successful aging,” and “non‐frailty” were often used interchangeably in the literature. However, a comparative analysis based on attributes of robustness confirmed these are distinct concepts, leading to their classification as related terms. “Intrinsic capacity” and “resilience” were also identified as a related term. To clarify conceptual boundaries, this study examined whether each of these related concepts fully aligned, partially aligned, or did not align with the three identified attributes (Table 3).

**TABLE 3:** Comparison of robustness in older adults and related terms.

Related terms	Attributes of robustness in older adults		
	Multidimensional vitality	Resilient stability	Functional autonomy
Successful aging	●	◐	●
Non‐frailty	◐	○	○
Intrinsic capacity	●	○	○
Resilience	○	◐	○

*Note:* ● Fully Aligns: The related term fully aligns with the specified attribute of “Robustness in Older Adults.” It fundamentally and explicitly integrates the attribute into its definition; ◐ Partially Aligns: The related term partially aligns with the specified attribute of “Robustness in Older Adults.” The attribute is only partially reflected or indirectly incorporated into the definition of the related term; ○ Does Not Align: The related term does not explicitly align with the specified attribute of “Robustness in Older Adults.”

#### 3.5.1 Successful Aging

Successful aging is most widely recognized through Rowe and Kahn's model, which includes a low probability of disease and disability, high physical and cognitive functioning, and active engagement with life (Rowe and Kahn 1987, 1997). However, this model has been criticized for several limitations, such as the exclusion of older adults' subjective perspectives, Western cultural bias, and potentially discriminatory views toward individuals with disabilities (Katz and Calasanti 2015). Various alternative approaches have been proposed, including the Selective Optimization with Compensation (SOC) model (Baltes et al. 1999) and concept analysis of Flood (2002), with a growing emphasis on incorporating subjective experiences and cultural contexts. To date, numerous successful aging measurement tools have been developed (Tong et al. 2022), generally encompassing physical, psychological, and social domains. Depending on the theoretical framework and cultural context, these tools also explore diverse areas such as spirituality, economic stability, family relationships, life meaning, learning, and adaptive strategies (Chung and Park 2008; Feng and Straughan 2017; Lee et al. 2017).

While various definitions of successful aging exist (Martin et al. 2015), this study compared it with robustness based on Rowe and Kahn's (1997) model, which is most widely known and has abundant empirical evidence. Successful aging aligns with two attributes of robustness—multidimensional vitality and functional autonomy—but differs significantly from resilient stability. Whereas successful aging emphasizes achieving an ideal state through the prevention of disease and disability and maintaining high levels of functioning, robustness—particularly in terms of resilient stability—focuses on adaptive responses and recovery capacity in health‐threatening situations. Successful aging highlights the maintenance of optimal conditions, while robustness underscores adaptation and recovery in the face of challenges.

#### 3.5.2 Non‐Frailty

Frailty is defined as a clinically recognizable state of increased vulnerability resulting from age‐related decline in physiological reserves and functioning across multiple systems (Proietti and Cesari 2020) while non‐frailty refers to the absence of this condition. When compared to the three core attributes of robustness, non‐frailty partially aligns with multidimensional vitality. As frailty assessment has recently expanded beyond the physical domain to include cognitive and social dimensions (Bu et al. 2021; Shah et al. 2023), non‐frailty now implies the absence of deficits in multidimensional domains. However, this reflects only a passive state of “not being frail,” which differs from the active and positive vitality embodied in robustness. Moreover, non‐frailty does not align with the attributes of resilient stability or functional autonomy. In other words, non‐frailty represents a minimal baseline—simply the absence of problems—whereas robustness reflects a positive health state that exceeds this threshold.

#### 3.5.3 Intrinsic Capacity

Intrinsic capacity is defined as the composite of all physical and mental capacities of an individual (World Health Organization 2015). The World Health Organization (WHO) suggested that intrinsic capacity comprises five domains: cognition, locomotion, sensory (vision, hearing), psychological, and vitality (Gonzalez‐Bautista et al. 2020).

Intrinsic capacity aligns with the attribute of multidimensional vitality in robustness, as both comprehensively address individuals' fundamental capacities. However, intrinsic capacity focuses primarily on the inherent abilities themselves and does not directly capture what robustness emphasizes—namely, functional autonomy, which refers to the ability to perform daily activities and make personal decisions independently or with appropriate support, and resilient stability, the capacity to maintain stability through adaptation and recovery in the face of change and adversity.

#### 3.5.4 Resilience

Resilience is defined as the process of adapting to, negotiating, or recovering from significant adversity or stressors, and may ideally involve growth beyond the prior state of functioning (Resnick 2014; Windle 2011). Robustness and resilience represent fundamentally distinct constructs (Ukraintseva et al. 2016); robustness emphasizes resisting deviation from baseline functioning, whereas resilience emphasizes recovering after such deviation has occurred.

In terms of alignment with the three attributes of robustness, resilience partially overlaps with resilient stability, as both involve adaptive responses to challenges and stressors. However, this overlap is only partial, as resilience is primarily conceptualized as a reactive process triggered after adversity, whereas robustness reflects a broader, more proactive state of health. Furthermore, resilience does not encompass multidimensional vitality or functional autonomy, which are central to robustness. Therefore, resilience is best understood as a related but conceptually distinct term.

### 3.6 Model Case

Hana is a 77‐year‐old woman who maintains an active lifestyle across physical, emotional, cognitive, and social dimensions. She swims and does yoga three times a week, walks daily, participates in a weekly book club, and learns Spanish through an online app. She volunteers in a local group, organizing cultural events each month, and maintains a positive outlook with low levels of anxiety and depression.

Last year, Hana underwent coronary artery bypass graft (CABG) surgery following a diagnosis of severe cardiovascular disease. She actively engaged in rehabilitation, adopted new dietary and exercise habits, and used a wearable heart rate monitor to track her recovery. With initial family support, she gradually transitioned to independently managing her daily life.

Hana independently performs ADLs and IADLs, prepares her own meals, and manages household chores while knowing when to seek assistance. She makes her own healthcare decisions, recently consulting her doctor to adjust her hypertension treatment plan and maintains financial independence. She has also discussed future living arrangements with her family in preparation for aging in place.

In this case, Hana demonstrates all three attributes of robustness. She exhibits vitality across physical, emotional, cognitive, and social dimensions, demonstrates the capacity to adapt and recover in the face of serious illness, and maintains independence in daily life and decision‐making.

### 3.7 Borderline Case

Jun is a 72‐year‐old man living with his adult children. He walks daily and maintains age‐appropriate cognitive function, but has recently reported lethargy and mild depressive symptoms, spending most of his time at home with minimal social engagement.

Diagnosed with type 2 diabetes and chronic kidney disease 3 years ago, he takes five medications. While adherent to his prescriptions, he does not engage in proactive self‐management, and recent check‐ups revealed deterioration in blood glucose and kidney function. Following hip fracture surgery last year, poor rehabilitation participation has left his walking function below pre‐surgical levels.

Jun performs basic ADLs independently but relies on his children for some IADLs, including grocery shopping and transportation. Financial decisions, such as managing bills and medical expenses, are fully delegated to his children.

In this case, Jun partially meets all three attributes of robustness. Physical and cognitive vitality are preserved, but emotional and social vitality are diminished; chronic conditions have worsened and surgical recovery has been delayed; and basic ADLs are maintained but with considerable dependence on his children for IADLs and financial decision‐making.

### 3.8 Contrary Case

Soo is a 78‐year‐old woman residing in a long‐term care facility. Moderate Alzheimer's disease has severely impaired her cognitive function, and she frequently fails to recognize those around her with little to no social interaction. Chronic heart failure and osteoarthritis make even basic physical activity difficult, and persistent pain and fatigue leave her withdrawn and lethargic.

She has more than five chronic conditions and takes eight medications but is unable to manage them independently due to cognitive decline, with care staff assuming full responsibility. Over the past 6 months, she has been hospitalized twice with pneumonia, failing to recover to her prior functional level each time, with her overall status continuing to decline.

Soo requires full assistance for basic ADLs including eating, bathing, and mobility. She is unable to make independent decisions regarding her health or daily life, with all medical and financial decisions made by her family and healthcare team and participates in no social roles or productive activities.

In this case, Soo meets none of the three attributes of robustness. Physical, cognitive, emotional, and social vitality are all severely compromised; repeated hospitalizations have yielded no functional recovery, and independence in ADL performance, decision‐making, and social engagement has been completely lost.

## 4 Discussion

This study examined the concept of robustness in older adults using the evolutionary approach (Rodgers and Knafl 2000). Analysis of 31 key studies spanning approximately 30 years across gerontology, geriatrics, or gerontological nursing, with diverse racial and ethnic groups representation, supports the broad applicability of the concept across varied population groups.

This study identified three attributes of robustness in older adults. Through temporal analysis, this study traced the conceptual evolution of robustness from “successful aging” in the 1980s, to “physical non‐frailty” in the 2000s, and finally to more recent multidimensional approaches, resulting in the identification of multidimensional vitality, encompassing physical, emotional, cognitive, and social dimensions, as a core attribute. The interdisciplinary comparison further revealed that, unlike robustness in other academic fields which emphasizes immutable stability, robustness in older adults is defined by resilient stability, which is the ability to maintain stability through flexible adaptation and recovery in the face of challenges (Kim et al. 2023; Md Fadzil et al. 2023; Xu et al. 2021), aligning with contemporary perspectives that regard aging as a life stage with potential for continued adaptation and growth (Klusmann and Kornadt 2019; Morganti 2024). The third attribute, functional autonomy, extends beyond mere physical independence to encompass older adults' agency and choice. Recognizing that meaningful participation requires older adults to determine the level and manner of their own participation (Dizon et al. 2020), this attribute reflects a fundamental shift in perspective from viewing older adults as passive recipients to active agents in their own care, a principle emphasized in Age‐Friendly Care models (Millar et al. 2026).

The three attributes are not conceptually parallel. Multidimensional vitality represents the foundational state of robustness, whereas resilient stability and functional autonomy are the capacities that emerge from this state. At the same time, the exercise of these two capacities contributes to maintaining and reinforcing the state of multidimensional vitality, such that the three attributes form a mutually reinforcing and dynamic relationship. Furthermore, given that resilient stability and functional autonomy are capacities emerging from an inherently multidimensional state of vitality, we propose that these capacities may themselves manifest across physical, psychological, and social dimensions. As the evolutionary approach emphasizes, concepts are subject to continuous change across time and context (Rodgers and Knafl 2000). Future research may therefore advance the theoretical refinement of robustness by more systematically examining how these capacities manifest across multiple dimensions.

Among the antecedents of robustness in older adults identified in this study, beneficial lifestyle behaviors represent intra‐individual factors operating at the physiological and behavioral level, whereas socioeconomic resources and supportive social networks represent extra‐individual environmental factors that shape the broader context in which robustness develops (Minkler et al. 2000). These antecedents are distinct yet interconnected, collectively influencing robustness in older adults (González et al. 2022). For example, insufficient socioeconomic resources make it difficult to maintain healthy lifestyle behaviors (Xue et al. 2021), and without adequate social support (e.g., encouragement to exercise, maintain diet, or adhere to medication), sustaining such behaviors through individual effort alone becomes difficult (Thoits 2011). Accordingly, single‐level interventions are insufficient, reinforcing the importance of multilevel and integrated strategies in healthcare policies and health promotion programs for older adults (Kodner and Kyriacou 2000).

Meanwhile, the consequences of the robustness concept—enhanced quality of life, reduced healthcare needs, and decreased mortality—align with the core outcomes pursued by care services targeting older adults (Brown et al. 2013; Kaarlola et al. 2006; Kim et al. 2018). This demonstrates that the concept of robustness is not merely a theoretical construct but is closely linked to tangible health outcomes in real‐world care settings. Therefore, this study supports the notion that strengthening robustness may serve as an efficient means of achieving multiple health goals simultaneously (Chang et al. 2018; Liu et al. 2021; Stolz et al. 2024).

Several studies have identified associations between robustness and biomarkers such as serum SIRT6 levels, CD36 mRNA expression in peripheral blood mononuclear cells, and inflammatory cytokines including TNF‐α and CXCL10, suggesting their potential as indicators of robustness (Choi et al. 2019; Lee et al. 2022; Zhu et al. 2023). Biomarkers do not represent events that precede the emergence of a concept or result from its application; therefore, they were not considered antecedents or consequences of robustness in this study according to Rodgers and Knafl (2000). Nevertheless, the clear association between these biomarkers and robustness suggests that biomarkers may serve as useful tools for predicting and assessing robustness in older adults (Biomarkers of Aging Consortium et al. 2024) and underscores the need for their systematic use in future research on preventive geriatric care and in the development of standardized measurement tools for robustness.

In analyzing related terms to clarify conceptual boundaries, “health” was excluded due to its broad scope, which may hinder conceptual clarity and contextual relevance (Tofthagen and Fagerstrøm 2010), and its status as one of the four metaparadigm concepts in nursing (Bender 2018). Nevertheless, as robustness is oriented toward the health status of older adults (Rippl et al. 2025), the evolving concept of health may serve as a higher‐order criterion for evaluating the identified attributes. Contemporary perspectives have moved beyond the traditional notion of complete physical, mental, and social well‐being (World Health Organization 1948) toward a more dynamic understanding centered on adaptive capacity and functional ability. Huber et al. (2011) redefined health in terms of adaptive capacity and self‐management, and the World Health Organization defined healthy aging as the maintenance and enhancement of functional ability (World Health Organization 2017). This perspective aligns with the attributes of robustness identified in this study.

Focusing on individual older adults' robustness provides a foundation for personalized treatment (Snyderman 2012) that goes beyond traditional age‐based treatment decisions (Fraenkel et al. 2006; Neal et al. 2022). This approach enables differentiated treatment approaches based on robustness levels even within the same chronological age group (Chang et al. 2019; Mehrabi and Béland 2021; Rothrock et al. 2019; Stolz et al. 2024; Xu et al. 2021).

Despite the clinical significance of robustness, current practice continues to rely on frailty assessment tools that measure robustness only indirectly, capturing limited aspects of the concept (Chang et al. 2019; Ensrud et al. 2007; Kim et al. 2019). To implement robustness in clinical practice for older adults, the development of standardized assessment tools is essential. Such tools must be grounded in a clear conceptual definition of robustness, which this study provides.

### 4.1 Limitations

This study has several limitations. First, papers were limited to those published in specific academic databases, meaning relevant studies addressing robustness in older adults may have been excluded if not indexed in these databases. Second, gray literature was not included, potentially missing relevant studies outside indexed databases. Third, only English‐language studies were included, which may have excluded important research from non‐English speaking regions. These limitations may have restricted the comprehensiveness and diversity of perspectives.

## 5 Conclusion

This study clarified robustness in older adults as a concept characterized by three core attributes: multidimensional vitality, resilient stability, and functional autonomy. By grounding this conceptualization in systematic analysis of literature, this study offers a durable foundation that can anchor future measurement, intervention, and policy development in gerontological nursing. In the context of a rapidly aging population, the concept of robustness holds significant implications for both healthcare practice and research, offering a theoretically grounded basis for individualized geriatric care and future instrument development.

## 6 Relevance for Clinical Practice

This concept analysis provides a foundation for transforming geriatric healthcare practice by shifting from age‐ and deficit‐based assessments toward individualized, capability‐focused care. It directs clinical attention to the breadth and quality of older adults' remaining capabilities across multiple domains.

To implement this framework in practice, standardized assessment tools that operationalize all three attributes are needed to enable consistent, multidimensional evaluation across clinical settings. These tools should capture not only physical performance but also psychological resilience and social participation. Unlike existing frailty indices that provide a binary frail/non‐frail classification, robustness‐specific tools should assess the degree and profile of individual capabilities, supporting nuanced clinical decision‐making and improved care coordination across multidisciplinary teams.

This conceptual clarity enables differentiated treatment approaches within the same age cohort. Healthcare providers can apply more aggressive interventions for older adults with higher robustness, while adopting conservative, function‐maintaining strategies for those with lower robustness, particularly in complex decisions such as cancer treatment, surgical interventions, and rehabilitation planning. For nursing practice, this framework supports a shift from deficit‐focused care to proactive, robustness‐enhancing approaches across diverse care settings, including community and transitional care contexts (Xu et al. 2025).

## Author Contributions

**Yoosun Yang:** conceptualization, methodology, data curation, investigation, formal analysis, writing – original draft, writing – review and editing, validation, visualization. **Jun‐Ah Song:** supervision, funding acquisition, validation, writing – review and editing, resources.

## Funding

This work was supported by the National Research Foundation of Korea (NRF) grant funded by the Korea government (Ministry of Science and ICT) (RS‐2024‐00340386).

## Ethics Statement

The authors have nothing to report.

## Conflicts of Interest

The authors declare no conflicts of interest.

## Supporting information


**Supporting Table 1.** Search Strategy. This table presents the detailed search strategy employed across four electronic databases (PubMed, CINAHL Complete, Embase, and Scopus) to identify relevant studies for this review.

## Data Availability

The data that support the findings of this study are available from the corresponding author upon reasonable request.
