# Supplementary material for: Robustness in Older Adults: A Concept Analysis Using Rodgers' Evolutionary Approach
Source: Nurs Health Sci. 2026 May 4;28:e70349. doi: 10.1111/nhs.70349 (PMC13138878; doi:10.1111/nhs.70349)
Supplement: Supplementary file 1 — Supporting Table 1. Search Strategy. This table presents the detailed search strategy employed across four electronic databases (PubMed, CINAHL Complete, Embase, and Scopus) to identify relevant studies for this review. [file NHS-28-e70349-s001.docx]

Supplementary Table 1. Search Strategy

| **Database** | **Search Query** | **Results (n)** |
| --- | --- | --- |
| PubMed | ((("robust old*"[Title/Abstract]) OR ("robust elder*"[Title/Abstract])) OR ("robust agi*"[Title/Abstract])) OR ("robust age*"[Title/Abstract]) | 287 |
| CINAHL Complete | AB "robust old*" OR AB "robust elder*" OR AB "robust age*" OR AB "robust agi*" | 81 |
| Embase | 'robust old*':ab,ti OR 'robust elder*':ab,ti OR 'robust agi*':ab,ti OR 'robust age*':ab,ti | 394 |
| SCOPUS | (TITLE-ABS-KEY("robust old*") OR TITLE-ABS-KEY("robust elder*") OR TITLE-ABS-KEY("robust agi*") OR TITLE-ABS-KEY("robust age*")) | 723 |
| **Total** |  | **1,485** |

Note. The search was conducted on 14th April 2024. No language or date restrictions were applied.
